# Connexin-43 can delay early recurrence and metastasis in patients with hepatitis B-related hepatocellular carcinoma and low serum alpha-fetoprotein after radical hepatectomy

**DOI:** 10.1186/1471-2407-13-306

**Published:** 2013-06-24

**Authors:** Zu-Sen Wang, Li-Qun Wu, Xin Yi, Chao Geng, Yu-Jun Li, Ru-Yong Yao

**Affiliations:** 1 Department of Hepatobiliary Surgery, Affiliated Hospital of Medical College Qingdao University, Qingdao, Shandong Province, 266003, China; 2 Department of Pathology, Affiliated Hospital of Medical College Qingdao University, Qingdao, Shandong Province, 266003, China; 3 Department of Central Laboratory, Affiliated Hospital of Medical College Qingdao University, Qingdao, Shandong Province, 266003, China

**Keywords:** Carcinoma, Hepatocellular, Hepatitis B, Alpha-fetoprotein, Cx43, VEGF, CD105, Early recurrence, Metastasis, Prognosis

## Abstract

**Background:**

We studied the relationships among Cx43, CD105, and VEGF in specimens of hepatitis B virus (HBV)-related hepatocellular carcinoma (HCC) with different serum AFP levels with respect to recurrence and metastasis.

**Methods:**

Expressions of Cx43, CD105, and VEGF in 234 HCC tissue specimens were examined using tissue microarray and immunohistochemistry. *Cx43* mRNA expression was examined in 38 frozen HCC specimens using RT-PCR. Correlations between these expressions and tumor recurrence, metastasis, and prognosis were analyzed using Kaplan–Meier and Cox regression analyses.

**Results:**

Cx43 expression correlated with early tumor recurrence (*P* = 0.001), disease-free survival (*P* = 0.026), and overall survival (*P* = 0.000) in patients with serum AFP < 400 ng/ml, but not in those with serum AFP ≥ 400 μg/L. Cx43 expression is an independent predictor of later recurrence and longer overall survival and is inversely correlated with expression of CD105 and VEGF (*P* = 0.018 and 0.023, respectively), histological differentiation (*P* = 0.002), vessel tumor embolism (*P* = 0.029), and focal number (*P* = 0.017). Immunohistochemistry showed that Cx43 expression in patients with low AFP was lower in patients with distant metastases than in those with no metastasis or those with liver metastasis. Patients with early recurrence expressed less *Cx43* mRNA than did those with no recurrence (χ^2^ = 9.827, *P* = 0.002).

**Conclusions:**

Cx43 expression can delay early HCC recurrence, metastasis, and poor prognosis after radical hepatectomy in patients with HBV-related HCC and low AFP.

## Background

Hepatocellular carcinoma (HCC) is the sixth most common cancer and the third most common cause of cancer death [1]. An estimated 748,300 new liver cancer cases and 695,900 cancer deaths occurred worldwide in 2008 [2]. Although an increasing number of new methods are now applied to treat these patients, surgery (including hepatectomy and liver transplantation) is still the most important therapeutic approach for patients with HCC [3]. Transplantation is the preferred treatment option for small and resectable HCC [4], but its use is limited by the lack of donor organs [5]. Although appropriate selection of patients with HCC increases the clinical survival benefit after radical liver resection [6], the prognosis of HCC remains poor because of the high rate of recurrence and metastases after radical resection [7,8]. A primary research focus involves how to decrease the HCC recurrence and metastasis rates worldwide.

Chronic hepatitis B virus (HBV) and hepatitis C virus (HCV) infections induce approximately 75–80% of HCC in the world [9]. Chronic HBV infection accounts for about 60% of the total liver cancer in developing countries and about 23% of the cancer in developed countries. This infection results in approximately one-third of all cases of liver cirrhosis and more than three-quarters of all cases of HCC worldwide [10]. Serious endemicity of HBV infection is present in China. In a literature review, Han et al. [11] reported that a high serum viral load is the most reliable indicator of viral replication in predicting development of HCC (serum viral load of ≥10^4^ copies/mL for HCC occurrence and 10^4^ copies/mL for a poor prognosis). In addition, HBV genotype C is closely associated with HCC in cirrhotic patients aged ≥ 50 years, whereas genotype B is associated with development of HCC in noncirrhotic young patients (< 50 years) and postoperative relapse of HCC. Thus, we confined our investigation of HCC to patients with HBV-related HCC (HBV-HCC) to reduce possible confounding variables, and we plan to study the relationship between and mechanisms of early recurrence of HCC and HBV-related factors (e.g., serum HBV viral load and HBV genotype and mutations).

Connexin-43 is a member of the connexin (Cx) protein family, which forms the basis of gap junctions (GJs). GJs provide a medium through which GJ intercellular communication (GJIC) is expressed; GJs are largely formed by Cx proteins. In liver tissue, GJIC mainly involves three kinds of Cx—Cx26, Cx32, and Cx43—whose distribution depends on cell type and location in the liver lobule [12]. HCC cells typically express high levels of Cx43, low levels of Cx32, and no Cx26. Cx43, which is clearly expressed in HCC cells, is mainly located in the cytoplasm; a small amount is located in areas connecting nonadjacent cells on the cell membrane, which disrupts GJIC [13].

In this retrospective study, the protein expressions of Cx43, VEGF, and CD105 in HCC tissue with different serum AFP levels were examined by tissue microarray and immunohistochemical staining; expression of *Cx43* mRNA was examined by RT-PCR to explore the relationship between Cx43 and early recurrence, metastasis, and prognosis in patients with HCC after radical resection.

## Methods

### Patients

Samples were taken from 249 patients with HBV-HCC who underwent radical resection from January 2003 to December 2008 at the affiliated hospital of the Medical College of Qingdao University. The study protocol was approved by the Ethics Committee of the Qingdao University. All patients signed an informed consent form and met the medical ethics requirements. Of these, 15 who underwent preoperative transcatheter arterial chemoembolization were excluded; of the remaining 234 patients, 199 were male and 35 were female, with an average age of 54.2 years (range: 15–82 years). According to the TNM classification (AJCC 7th edition, 2009), 137 patients were at Stage I, 30 were at Stage II, 63 were at Stage III, and 4 were at Stage IV. Control specimens were from adjacent noncancerous tissue within 2 cm from the incisal margin (81 cases), and intraoperative biopsies were from patients with portal hypertension (79 cases). Specimens from 38 patients with HBV-HCC who underwent radical resection from January 2011 to June 2011 were frozen by liquid nitrogen immediately and cryopreserved at ^–^80°C.

#### Tissue microarray and immunohistochemistry

A tissue arrayer (HT-1,beijing,china) was used to make 42 (6 × 7) 4-μm-thick lattice arrays, which were first stained with hematoxylin/eosin to confirm the tissue type and then immunohistochemically stained using PV-6000 methods (two steps), following the manufacturer’s instructions. Briefly, PBS replaced the primary antibody as the negative control; positive tissues were used as positive controls. Rabbit anti-human Cx43, VEGF, and CD105 monoclonal antibodies as well as immunohistochemical detection reagents were purchased from ZSGB-Bio Company (Beijing, China).

#### RT-PCR

RNAiso Plus and the PrimeScript RT-PCR Kit were produced by Takara Dalian (Dalian, China). PCR Amplifier was produced by Eppendorf Company (Hamburg, Germany). The gel electrophoresis apparatus was produced by VILBER LOURMAT Company (Marne-la-Vallee, France). Primers were designed using Primer 3 and synthesized by BGI Company (Shenzhen, China). Primer sequences of Cx43 were as follows: forward: 5′-GGCTGCTCCTCACCAACGGCC-3′; reverse: 5′-AGGTCATCAGGCCGAGGTCTG-3′. Total RNA isolation was performed according to the instructions of RNAiso Plus; reverse transcription was performed according to instructions of the PrimeScript RT-PCR Kit with a total volume of 20 μl. The PCR reaction took place in a total volume of 20 μl using cDNA 0.5 μl, forward primer 0.5 μl, reverse primer 0.5 μl, Premix TAQ 12.5 μl, and RNA-free H_2_O 6 μl. PCR was performed at 95°C for 5 min; followed by 37 cycles of 95°C for 30 s, 58°C for 30 s, and 72°C for 35 s; and a final extension of 72°C for 10 min. Amplified products of Cx43 were 332 bp long.

#### Immunohistochemistry

Two pathologists graded the specimens in a double-blind manner. Brown granules in the cytoplasm were considered positive for Cx43, and those in the cell membrane were considered positive for CD105; 100 tumorous and nontumorous hepatic cells were counted in each high-power field, and 5 fields were observed for each array. The average percentages of positive cells were then calculated. A specimen was considered Cx43^+^ if ≥ 5% of cells were positive. The CD105 microvessel density (MVD) refers to the Weidner method [14]. A specimen was considered VEGF^+^ if ≥ 50% of cells were positive. Cells were considered CD105^+^ if they were beyond the MVD-CD105 mean line.

#### PCR products

We assessed the amplified RT–PCR products by electrophoresing 10 μl in a 2% agarose gel containing ethidium bromide (0.5 mg/ml) in 0.04 M Tris-acetate and 0.001 M EDTA (TAE) buffer at 120 V for 30 min. Gels were then developed by a UV transilluminator, and the results were scanned by a computer.

#### Follow-up

Patients were followed up monthly for 3 months and every 3 months thereafter. The blood alpha-fetoprotein (AFP) level, liver function, abdominal ultrasound or CT, and chest CT were monitored at each follow-up. Patients who did not come in for appointments received follow-up calls and were followed until 31 December 2011 or until death. Tumors were considered to have recurred based on their appearance by imaging examination (abdominal ultrasound, CT, or MRI); hepatic arteriography or biopsy was used in unclear cases. Early recurrence was defined as recurrence within 1 year.

#### Statistical analysis

All data were analyzed using SPSS statistical software (ver. 13.0; SPSS Inc., Chicago, IL). Categorical variables were compared by the χ^2^ test and Pearson’s correlation analysis. Survival analysis was calculated with the Kaplan–Meier method and compared using the log-rank test. Multivariate analyses were performed using a Cox proportional hazards model to identify independent prognostic factors. A *P* value of < 0.05 was considered statistically significant.

## Results

### Survival analysis

The clinicopathologic characteristics of 234 patients with HBV-HCC are summarized in Table 1. A total of 159 (67.9%) patients had a serum AFP level of < 400 μg/l (low AFP) and 75 (32.1%) had a serum AFP level of ≥ 400 μg/l (high AFP). The disease-free survival (DFS) for the low AFP and high AFP groups was 31.64 and 13.8 months, respectively (χ^2^ = 4.403, *P* = 0.036), and the overall survival rate (OS) was 84.0 and 58.55 months, respectively (χ^2^ = 2.588, *P* = 0.108). The early recurrence rate was 27.0% (43 cases) and 49.3% (37 cases), in the low AFP and high AFP groups respectively (χ^2^ = 11.253, *P* = 0.001).

**Table 1:** **Clinicopathological characteristics of 234 patients with HBV**-**related HCC**

**Characteristics**	**Result**		
	**All cases**	**Cx43**^**+ **^**Group**	**Cx43**^**- **^**Group**
Gender (male/female)	199/35	90/9	109/26
Mean age (range), years	54.2(15–82)	55.1(24–82)	55.5(15–74)
Preoperative serum AFP (<400/≥400 ng/mL)	159/75	78/21	81/54
Tumor size (≤ 5/>5 cm)	116/118	52/47	64/71
Edmondson-Steiner classification (I/II/III/ςςς)	25/134/11/64	15/64/4/16	10/70/7/48
Foci number (1/>1)	203/31	79/20	124/11
Liver capsule invasion (yes/no)	163/71	62/37	101/34
Satellite foci (yes/no)	31/203	13/86	18/117
TNM stage (I/II/III/ςςς)	137/30/63/4	58/17/23/1	79/13/40/3
Cirrhosis (yes/no)	213/21	88/11	125/10
Vascular tumor thrombosis (yes/no)	26/208	9/90	17/118
Child-Pugh class (A/B)	228/6	98/1	130/5
ALB (>35/≤35 g/L)	215/19	92/7	123/12
Tbil ()	193/41	85/14	108/27
ALT (≤60/>60 U/L)	165/69	78/21	87/48
AST (≤42/>42 U/L)	157/77	74/25	83/52
1-year recurrence (yes/no)	80/154	24/75	56/79

### Cx43, CD105, and VEGF expression in HBV-HCC tissues and adjacent or cirrhotic tissues

**Figure 1:**
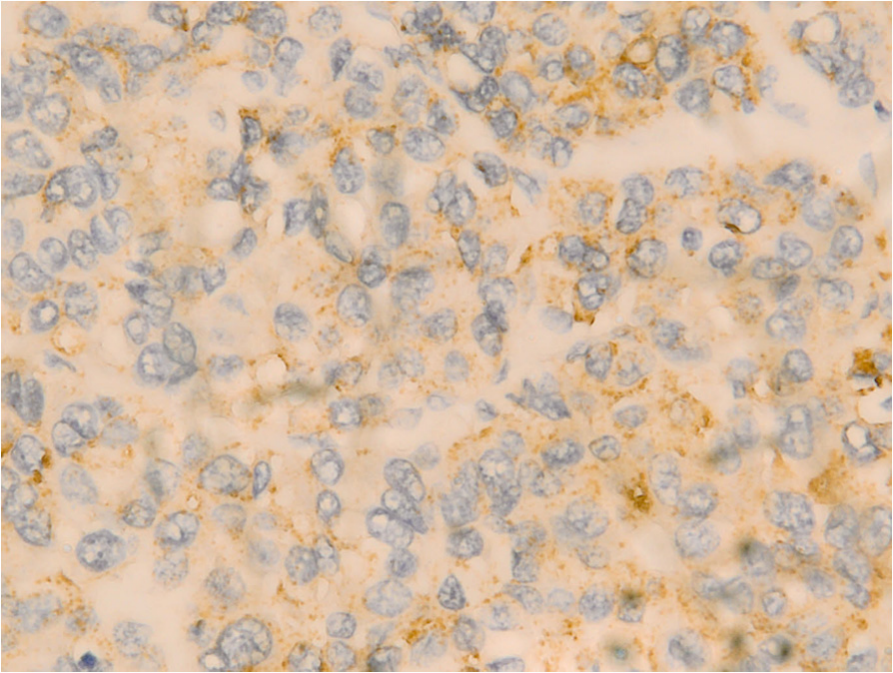
**Positive expression of Cx43 in HCC tissues (400×).**

Cx43 appeared in the cytoplasm as brown granules. The total Cx43^+^ rate was 42.3% (99/234) in HCC tissues (Figure 1), 72.8% (59/81) in adjacent tissues, and 92.4% (73/79) in cirrhotic tissues.

The Cx43^+^ rate was 49.1% (78/159) in the low-AFP group and 28% (21/75) in the high-AFP group, showing a significant difference (χ^2^ = 9.257, *P* = 0.002).

**Figure 2:**
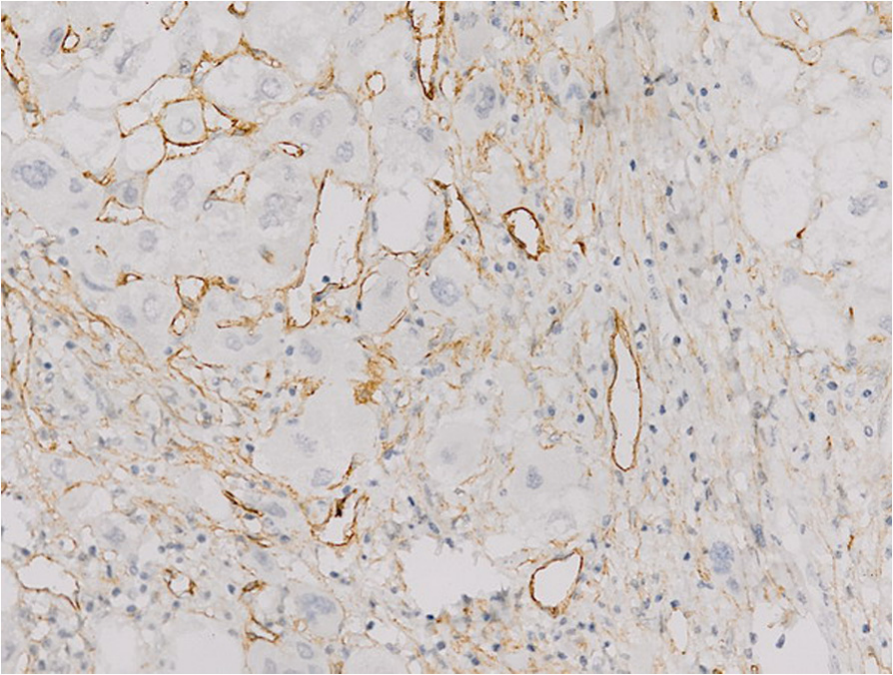
**Positive expression of CD105 in HCC tissues (400×).**

**Figure 3:**
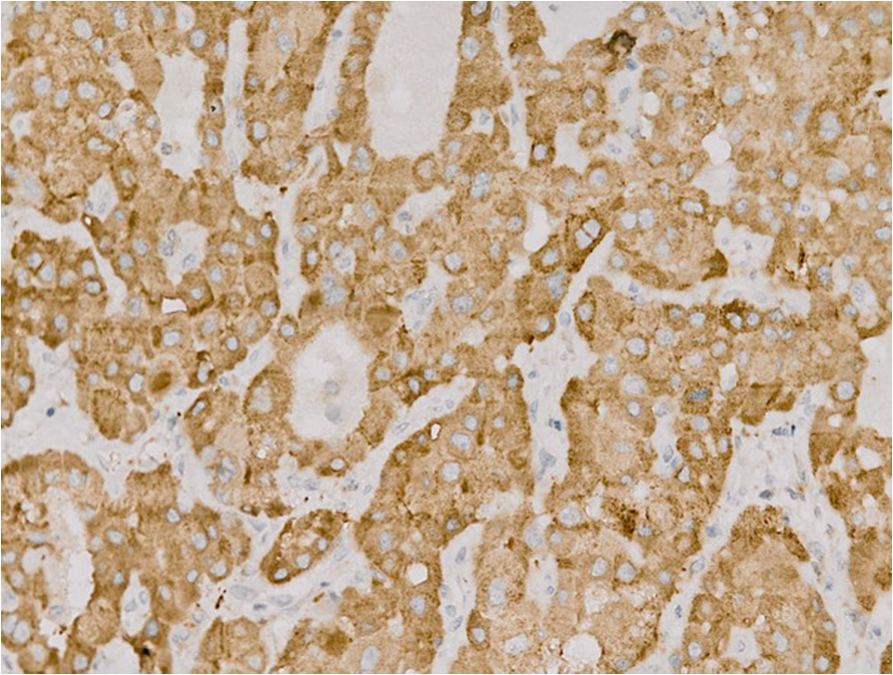
**Positive expression of VEGF in HCC tissues (400×).**

CD105 and VEGF were partly expressed in the cytoplasm as brown granules (Figures 2 and 3). The median MVD-CD105 was 19.0, and the positive CD105^+^ rate was 35.8% (57/159). The VEGF^+^ rate was 88.7% (141/159).

**Figure 4:**
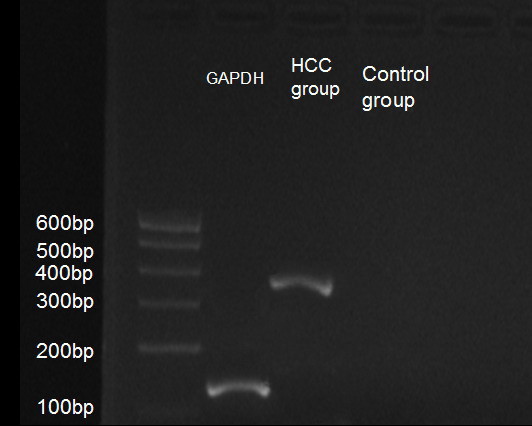
Expression of Cx43 mRNA in HCC tissues.

The 38 HCC specimens preserved in ^–^80°C were analyzed by RT-PCR, which showed a *Cx43*^+^ mRNA expression rate of 57.9% (22/38) (Figure 4).

### Relationship among serum AFP level, Cx43 expression, and patient prognosis

Kaplan–Meier analysis (log rank test) showed that although the DFS of the Cx43^+^ group was higher than that of the Cx43^–^ group (medians: 33.57 and 19.36 months, respectively), there was no statistical significance. The OS of the Cx43^+^ group was significantly higher than that of the Cx43^–^ group (*P* = 0.023). Cx43^-^ expression in patients with HCC with a low AFP level was significantly associated with a high early recurrence rate and poor prognosis (Table 2), unlike patients with HCC with a high AFP level. These results indicate that for patients with HCC with a low AFP level, Cx43^-^ expression is a likely predictor of early recurrence and a poor prognosis.

**Table 2:** **Correlation of high and low serum AFP levels and Cx43 expression with prognoses of patients with HBV**-**HCC who underwent radical resections**

	**Cx43 expression**	**n**	**DFS**			**OS**		
			**Median (m)**	**χ**^**2**^	***P *Value**	**Median (m)**	**χ**^**2**^	***P *Value**
Low AFP	+	78(23.3%)	44.00	4.963	0.026*	84.00	14.148	0.000*
	-	81(34.6%)	20.12			42.74		
High AFP	+	21(9.0%)	11.45	1.721	0.190	54.03	0.012	0.912
	-	54(23.1%)	16.80			61.99		
Total	+	99(42.3%)	33.57	2.440	0.118	84.00	10.657	0.001*
	-	135(57.7%)	19.36			47.97		

*P < 0.05.

### Cx43 and clinicopathological features among patients with a low AFP level

Chi-square tests showed that Cx43 expression was significantly related to histological differentiation (*P* = 0.023), multiple foci (*P* = 0.017), vascular tumor thrombosis (*P* = 0.001), and early recurrence (*P* = 0.006) (Table 3).

**Table 3:** **Relationship between Cx43 expression and clinicopathological factors in patients with HBV**-**HCC and low AFP levels**

		**Cx43**		**χ**^**2**^	***P *Value**
		**- (%)**	**+ (%)**		
Gender	Male	68(42.3%)	71(44.6%)	1.809	0.179
	Female	13(8.2%)	7(4.4%)		
Age	> 60y	18(11.3%)	27(17.0%)	3.008	0.083
	≤ 60y	63(39.6%)	51(32.1%)		
Edmondson-Steiner classification	I–II	51(32.1%)	66(41.5%)	9.969	0.002*
	III–IV	30(18.9%)	12(7.5%)		
Foci	Single	64(40.3%)	72(45.3%)	5.68	0.017*
	Multiple	17(10.7%)	6(3.7%)		
Tumor size	> 5cm	41(25.8%)	48(30.1%)	1.923	0.166
	≤ 5cm	40(25.2%)	30(18.9%)		
Liver capsule invasion	No	24(15.1%)	32(20.1%)	2.262	0.133
	Yes	57(35.8%)	46(28.9%)		
Satellite foci	No	73(45.9%)	73(45.9%)	0.636	0.425
	Yes	8(5.0%)	5(3.2%)		
Cirrhosis	No	4(2.5%)	10(6.3%)	3.075	0.080
	Yes	77(48.4%)	68(42.8%)		
Vascular tumor thrombosis	No	75(47.1%)	78(49.1%)	8.243	0.029*
	Yes	6(3.8%)	0(0%)		
Microvascular invasion	No	21(27.3%)	30(40%)	5.422	0.020
	Yes	18(23.4%)	8(10.3%)		
Child-Pugh class	A	78(49.1%)	78(49.1%)	4.102	0.246
	B	3(1.8%)	0(0%)		
TNM Stage	I–II	79(49.7%)	78(49.1%)	1.857	0.405
	III–IV	22(13.8%)	12(7.6%)		
1-year recurrence	No	50(31.4%)	66(41.5%)	7.544	0.006*
	Yes	31(19.5%)	12(7.6%)		

*P < 0.05. The HBV-HCC database of microvascular invasion was from 1 January 2008 to 31 December 2008; 87 patients entered the statistical analysis.

### Expression of Cx43 protein and metastasis location with low AFP level

Among patients with HBV-HCC and a low AFP level, the Cx43^+^ rate in the group with distant metastases was lower than that in patients with no metastases or liver-only metastases (both *P* < 0.05) (Table 4).

**Table 4:** **Relationship between Cx43 expression and recurrence location in patients with HBV**-**HCC and serum AFP of** <**400 μg**/**l**

	**Cx43**		**χ**^**2**^	***P *Value**
	**–**	**+**		
No recurrence	329(20.1%)	37(23.3%)	1.017	0.313
Recurrence	49(30.8%)	41(25.8%)		
No recurrence	32(23.9%)	37(27.6%)	0.042	0.838
Intra-hepatic recurrence	29(21.6%)	36(26.9%)		
No recurrence	32(34.0%)	37(39.4%)	8.393	0.004
Extra-hepatic metastasis	20(21.3%)	5(5.3%)		
Intra-hepatic recurrence	29(32.2%)	36(40%)	9.115	0.003
Extra-hepatic metastasis	20(22.2%)	5(5.6%)		

### Cx43 mRNA expression and early recurrence with low AFP level

*Cx43* mRNA was analyzed in 38 specimens from patients with HBV-HCC and a low AFP level, whose early recurrence rate was 26.7%; of these specimens, the *Cx43* mRNA^+^ rate was 57.9%. The early recurrence rate was lower in the *Cx43* mRNA^+^ group than in the Cx43 mRNA^–^ group (χ^2^ = 9.827, *P* = 0.002) (Table 5).

**Table 5:** **Relationship between *Cx43 *mRNA expression and early recurrence in patients with HBV**-**HCC and serum AFP of** <** 400 μg**/**l**

	***Cx43 *mRNA**		**χ**^**2**^	***P *Value**
	**+**	**–**		
Non recurrence	19(50%)	6(15.8%)	9.827	0.002
Early recurrence	3(7.9%)	10(26.3%)		

### Relationship between Cx43 expression and VEGF and CD105 expression

Expression of MVD-CD105 and VEGF was lower in the Cx43^+^ group than in the Cx43^–^ group in HCC specimens with a low AFP level (both *P* < 0.05) (Table 6).

**Table 6:** **Relationship among expressions of MVD**-**CD105**, **VEGF**, **and Cx43 in HCC specimens with serum AFP of** <** 400 μg**/**l**

	**Cx43**		***F *value**	***P *value**
	**+**	**–**		
MVD-CD105	19.36	25.84	5.184	0.018
VEGF + mean	76.38	80.00	5.309	0.023

### Expression of Cx43 protein and prognosis with a low AFP level

**Figure 5:**
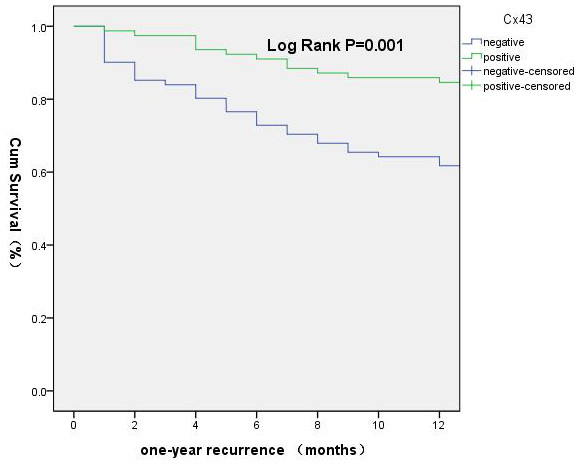
**Early recurrence disease**-**free survival curves.**

**Figure 6:**
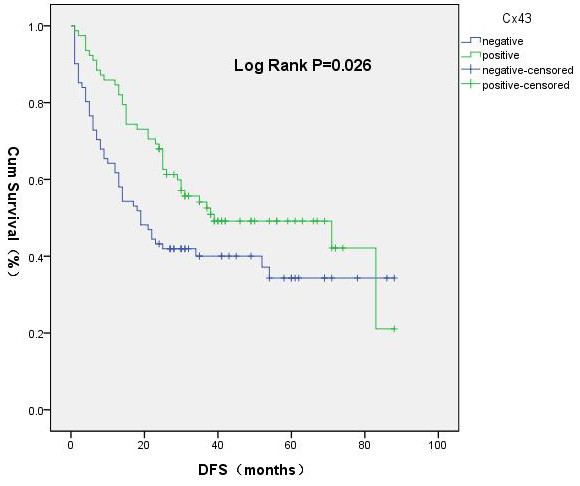
**Disease**-**free survival curves.**

**Figure 7:**
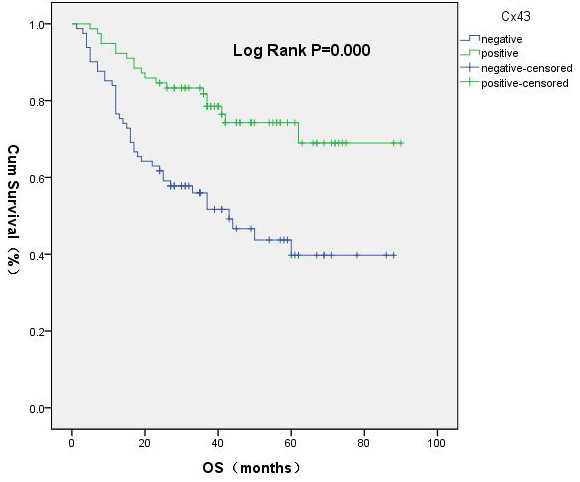
Overall survival curves.

In patients with HBV-HCC and a low AFP level, the early recurrence rate in the Cx43^+^ group was significantly lower than that in the Cx43^–^ group (14% and 36%, respectively; χ^2^ = 10.96, *P* = 0.001). The median DFS in the Cx43^+^ group (44.0 months) was longer than that in the Cx43^–^ group (20.1 months; χ^2^ = 4.963, *P* = 0.026) (Figures 5 and 6). The median OS in the Cx43^–^ group (42.7 months) was significantly shorter than in the Cx43^+^ group (84 months; χ^2^ = 14.15, *P* = 0.000) (Figure 7). Cox regression analysis showed Cx43^+^ expression to be an independent predictor of later recurrence (*P* = 0.042) and longer survival (*P* = 0.028) (Table 7).

**Table 7:** Cox regression analysis

		**RR**	**95**.**0% CI**	***P *Value**
One-year recurrence	Histological differentiation	2.762	1.482–5.147	0.001
	Cx43 expression	0.485	0.241–0.974	0.042
	Vascular tumor thrombosis	6.453	2.355–17.68	0.000
Disease-free survival	Histological differentiation	1.895	1.200-2.993	0.006
	Vascular tumor thrombosis	9.128	3.767-22.120	0.000
Overall survival	Histological differentiation	2.727	1.575–4.724	0.000
	Vascular tumor thrombosis	15.906	5.990–42.239	0.000
	Cx43 expression	0.515	0.285–0.930	0.028

## Discussion

Although surgical techniques have improved in recent years, the high recurrence rate of HCC after radical resection is an obstacle to survival. Previous research has shown that the 5-year survival rate of HCC can exceed 50% after radical resection. However, the tumor recurrence rate exceeds 70% at 5 years [3] and the small HCC recurrence rate is approximately 43.5% [15] despite the fact that the early occurrence rate is approximately 63.5% [16]. Our research showed an early recurrence rate of 32% and a 5-year recurrence rate of 61%.

In 1966, Loewenstein and Kanno first reported a relationship between GJs and cancer that established a lack of electrical coupling in rat hepatomas [17]. This phenomenon was observed in both chemically induced hepatomas and Morris and Novikoff’s transplanted hepatomas in rats [18,19].

Today, the hypothesis associating a lack of or diminished GJs with liver cancer is still valid [17] and continues to develop with new emerging concepts such as the possible involvement of stem cells and their GJIC capacity in carcinogenesis [20]. A literature review [17] described associations among how intercellular communication is involved in the carcinogenesis process, expression of connexin (including Cx26, Cx32, and Cx43), and particular stages of liver cancer progression by in vitro and in vivo data analyses.

Alpha-fetoprotein is synthesized by fetal hepatocytes; its seroprevalence in patients with HCC is nearly 70%. Because of its high specificity and convenient detection, AFP is a reliable index for screening, early diagnosis, and postoperative observation in patients with HCC. A low AFP level is also an important reference index for the CLIP score system. Because patients with HCC and a serum AFP level of ≥400 μg/l reportedly have significantly shorter survival times than patients with lower serum AFP levels [21], we used a serum AFP level of 400 μg/l as a boundary to divide patients with HCC into two groups: those with a high AFP level and those with a low AFP level.

We found that in patients with a low AFP level, Cx43^+^ tumor tissues were an independent predictor of later recurrence and a better prognosis. However, there was no relationship between Cx43^+^ expression and postoperative recurrence or prognosis in patients with a high AFP level. xTherefore, Cx43^+^ expression is an important predictor of later recurrence and a better prognosis in patients with a low AFP level who undergo radical hepatectomy.

Our results also showed that although Cx43 is mainly expressed in HCC cytoplasm (49.1%), this expression is lower than in paracancerous tissue (72.8%) and cirrhosis tissue (92.4%). We also found that *Cx43* mRNA is expressed in HCC tissue using the RT-PCR method.

Ma et al. [22] investigated 61 cases of HCC and 14 cases of normal liver tissues by immunohistochemical and in situ hybridization methods. The results indicated that the aberrant location of Cx43 protein could be responsible for the progression of hepatocarcinogenesis, and the mechanism may involve defects of Cx genes in post-translational processing. Ionta et al. used *Cx43* cDNA transfection to culture rabbit HCC cells *in vitro* and discovered that Cx43 reduced the multiplication capacity of these cells and restrained cancer cell growth [23]. Ogawa et al. established six rat HCC cell lines that exhibited different metastatic potentials after inoculation into the tail veins of nude mice and transfection of an siRNA targeting *Cx43* (as shown by cDNA array analysis), indicating that suppression of Cx43 expression in tumor cells reduced *in vitro* migration and invasion capacity and *in vivo* metastatic ability [24].

Our results also showed that Cx43 expression in HCC tissue is inversely related to invasion and metastasis, expression of VEGF and CD105, and vascular tumor thrombosis. Cx43 is an independent predictor of later recurrence and longer OS after HCC radical hepatectomy. The RT-PCR results also showed that expression of *Cx43* mRNA is related to later recurrence.

CD105-MVD reflects new vessel growth in HCC tissue and is an independent predictor of recurrence, metastasis, and vessel invasion in postoperative patients with HCC [25]. VEGF can substantially accelerate division and growth of vascular endothelial cells and angiogenesis. High VEGF levels can promote plasma protein exosmosis to induce fibrin deposition and new vessel generation, allowing invasion and metastasis; patients with HCC and higher VEFG expression in serum and HCC tissue have a poorer prognosis [9,26].

Immunohistochemistry results showed higher expression of CD105 and VEGF in patients with lower Cx43 levels. The reason may be that impaired GJIC function is closely related to early tumor occurrence, invasion, and metastasis [17]. Cx43 expression in HCC tissue can improve intercellular GJIC and restrain expression of the tumor accelerator angiogenesis factor, thus reducing recurrence and metastasis. This result indicates that Cx43 expression in HCC tissue is inversely related to important factors of postoperative HCC recurrence, such as tumor differentiation (*P* = 0.002) and vascular tumor thrombosis (*P* = 0.029), showing that Cx43 affects HCC recurrence and metastasis.

The present results indicate that patients with Cx43^+^ HBV-HCC have a longer OS than do patients with Cx43^–^ HBV-HCC. In particular, patients with Cx43^+^ HBV-HCC have better 1-year DFS and OS than do those with Cx43^–^ HBV-HCC and low serum AFP levels.

## Conclusion

This study demonstrated that Cx43^+^ expression in HBV-HCC tissue is a predictor of lower early recurrence rates and better prognosis in patients with low serum AFP levels and could be significant in terms of improving individualized treatments.

## Abbreviations

HCC: Hepatocellular carcinoma; Cx43: Connexin-43; GJs: Gap junctions; GJIC: Gap junction intercellular communication; HBV: Hepatitis B virus; VEGF: Vascular endothelial growth factor; RT-PCR: Reverse transcription polymerase chain reaction; AFP: Alpha-fetoprotein; MVD: Microvessel density; CT: Computed tomography; MRI: Magnetic resonance imaging; ALB: Serum albumin; Tbil: Total bilirubin; ALT: Alanine transaminase; AST: Glutamic-oxalacetic transaminase; DFS: Disease-free survival; OS: Overall survival; HBV-HCC: Hepatitis B-related hepatocellular carcinoma; High AFP: Serum AFP of ≥400 μg/l; Low AFP: Serum AFP of <400 μg/l

## Competing interests

The authors declare that they have no competing interests.

## Pre-publication history

The pre-publication history for this paper can be accessed here:

http://www.biomedcentral.com/1471-2407/13/306/prepub
